# HEALTH PROFESSIONALS’ ATTITUDES TOWARD TECHNOLOGY AND ARTIFICIAL INTELLIGENCE: AN EXPLORATORY STUDY

**DOI:** 10.1093/geroni/igad104.0267

**Published:** 2023-12-21

**Authors:** Moon Choi

**Affiliations:** Korea Advanced Institute of Science and Technology, Daejeon, Republic of Korea

## Abstract

The growing number of older adults, particularly those living alone, demands innovative health and social care solutions for the aging population. Mobile apps and other technologies are emerging every year to enhance the efficiency and effectiveness of geriatric health professionals’ work management. However, limited knowledge exists regarding geriatric health professionals’ perceptions of technology. The acceptance of technology is critical because emerging technologies cannot be fully utilized in the field without a positive attitude towards accepting them. The current study aims to investigate geriatric health professionals’ perceptions and attitudes towards technology and artificial intelligence (AI). The data were collected from a national survey of health professionals specialized in home visits conducted by community health centers in South Korea (N=1,026). Technology acceptance was evaluated using the Technology Acceptance Model Questionnaire, and study participants were asked about their self-reported knowledge of AI. A series of regression models showed that younger age, higher education level, and male gender were positively associated with higher technology acceptance levels. Regarding AI knowledge, only younger age was positively associated with better AI knowledge. The findings suggest the existence of a cohort difference in perceptions and attitudes towards technology among geriatric health professionals, which warrants further investigation into the underlying mechanism.

